# Pharmacology of Cell Adhesion Molecules of the Nervous System

**DOI:** 10.2174/157015907782793658

**Published:** 2007-12

**Authors:** Darya Kiryushko, Elisabeth Bock, Vladimir Berezin

**Affiliations:** Protein Laboratory, Department of Neuroscience and Pharmacology, Panum Institute Bld. 6.2, Blegdamsvej 3C, DK-2200, Copenhagen N, Denmark

**Keywords:** Recombinant, peptide, ligand, NCAM, cadherin, L1, pharmacology.

## Abstract

Cell adhesion molecules (CAMs) play a pivotal role in the development and maintenance of the nervous system under normal conditions. They also are involved in numerous pathological processes such as inflammation, degenerative disorders, and cancer, making them attractive targets for drug development. The majority of CAMs are signal transducing receptors. CAM-induced intracellular signalling is triggered *via* homophilic (CAM-CAM) and heterophilic (CAM - other counter-receptors) interactions, which both can be targeted pharmacologically. We here describe the progress in the CAM pharmacology focusing on cadherins and CAMs of the immunoglobulin (Ig) superfamily, such as NCAM and L1. Structural basis of CAM-mediated cell adhesion and CAM-induced signalling are outlined. Different pharmacological approaches to study functions of CAMs are presented including the use of specific antibodies, recombinant proteins, and synthetic peptides. We also discuss how unravelling of the 3D structure of CAMs provides novel pharmacological tools for dissection of CAM-induced signalling pathways and offers therapeutic opportunities for a range of neurological disorders.

## INTRODUCTION

Cell adhesion molecules (CAMs) are glycoproteins found on the cell surface that mediate cell-cell and cell-extracellular matrix (ECM) adhesion. CAMs can be divided into four classes: integrins, cadherins, members of the immunoglobulin (Ig) superfamily, and selectins. The first three classes are ubiquitously expressed in the nervous system. CAMs are not only 'mechanical' regulators of cell adhesion, but also signal transducing receptors. CAM-induced intracellular signalling is triggered *via* homophilic (CAM-CAM) and heterophilic (CAM - other counter-receptors) interactions resulting in a variety of cellular responses. These include neurite outgrowth and axonal pathfinding, synapse formation and remodelling, modulation of cell motility and survival, to mention a few. Because of this diversity CAMs not only play a pivotal role in the function of the nervous system under normal conditions, but also are involved in numerous pathological processes such as inflammation, neurodegeneration, and cancer. One strategy used to address CAM function is to study cells with genetic knock-out or knock-in of CAMs. However, it is not always possible to obtain the relevant constructs for a particular protein, and many animal systems are not easily amenable to genetic modifications. Therefore, it has become important to develop pharmacological tools that can target homophilic and/or heterophilic interactions of CAMs and thus modulate cellular responses induced by CAM binding.

This review discusses the progress in CAM pharmacology focusing on cadherins and CAMs belonging to the Ig superfamily, such as NCAM and L1 (see [178] for a detailed description of neural CAMs of the Ig-superfamily). We outline the structural basis of CAM-mediated cell adhesion and CAM-induced signalling. Different pharmacological approaches to study the functions of CAMs are presented, including the use of specific antibodies, recombinant proteins, and synthetic peptides. We also discuss how unravelling of the 3D structure of CAMs provides novel pharmacological tools for dissection of CAMs-induced signalling pathways and offers therapeutic opportunities for a range of neurological disorders.

## I. STRUCTURAL BASIS OF CAM-MEDIATED CELL ADHESION

### Cadherins

Cadherins are defined by the presence of the cadherin domain (CD) mediating Ca^2+^-dependent homophilic interaction. Calcium ions bind with the linker region that connects two CDs ensuring the rigid conformation of the cadherin molecules, which is a prerequisite of cadherin-mediated cell adhesion (Fig. (**1A**)). Based on the domain layout, cadherins are currently classified into seven subfamilies (see [167] for review). Among these, classic vertebrate cadherins were identified first as mediators of Ca^2+^-dependent adhesion in cultured cells and, independently, as regulators of morpho-genesis [162]. More recently, close attention was also drawn to protocadherins, which are only found in vertebrates and similarly to classic cadherins, can localize to synapses. These two cadherin subfamilies are most extensively characterized with regard to their structure and interaction partners (Fig. (**1A**), see [163] for details).

Classic cadherins bind to α*/*β, γ, and p120-catenins, which in turn interact with the actin cytoskeleton and the Rho family of GTPases inducing intracellular signalling and rearrangement of the cytoskeleton (reviewed in [194]). In addition, p120 can bind the non-receptor tyrosine kinases Fer and Fyn. These kinases phosphorylate Tyr142 of β-catenin, and this (unphosphorylated) tyrosine is necessary for the β-catenin/α-catenin association [119]. For protocadherins, only three interaction partners have been identified so far. First, the Pcdhα/CNR protocadherins interact with Fyn indicating that they also might play an important role in signalling [80]. Second, one of the splice variants of protocadherin 7 interacts with the α isoform of protein phosphatase 1 (PP1α, [195]). Third, the binding of protocadherin 18 to the phosphotyrosine-binding domain of the adaptor protein Disabled 1 (Dab1) has been reported [55].

Extracellularly, cadherins participate in heterophilic interactions with integrins [20] and the fibroblast growth factor receptor (FGFR, [185]), an interaction crucial for N-cadherin-induced neurite outgrowth (see Section III for details).

The mechanism of the cadherin-mediated homophilic interaction is still unclear (Fig. (**1B**)). Classic cadherins have been shown to form two types of dimers, namely *cis* (parallel association of molecules on the same plasma membrane) and *trans* (antiparallel association of molecules protruding from opposing cell membranes). Crystal structure studies of the CD1-CD2 module of N- and E-cadherins have shown that the formation of the *cis*-dimer is dependent on the presence of Ca^2+^, which stabilizes the CD chains and makes them resistant to proteolysis. Moreover, *cis*-dimers serve as blocks for further lateral clustering shown to be a prerequisite for stable cell adhesion [193]. The mechanism of *trans*-interaction of cadherins remains controversial (Fig. (**1B**)). Whereas initial studies only suggested an interaction between the two outmost domains of opposing N-cadherins, possibly with a zipper-like formation [147], more recent data suggest that the five CDs show variable degrees of lateral overlap [150]. Full lateral overlap of *trans*-dimers would imply a 20-25 nm distance between the opposing cell membranes, which is consistent with that found at adherens junctions. The classic cadherin-catenin complex is a principal component of the synaptic adherens junction. Protocadherins that localize to synapses include arcadlin and the Pcdhα/CNR protocadherins. However, their role in synaptic adhesion is not yet elucidated.

### L1-CAM

The L1-CAM (Fig. (**1A**)) belongs to the CAM subfamily also comprising NrCAM, neurofascin, axonin (ankyrin-binding glycoproteins), and the so-called close homolog of L1 (CHL1) (see [23] for details). L1-CAM participates in homophilic interactions and binds numerous heterophilic ligands including other CAMs (NCAM, TAG-1/axonin-1, contactin/F3/F11), proteoglycans (neurocan and phosphacan), ECM-associated molecules (laminin, tenascin), neuropilin-1, and receptor tyrosine phospatases (reviewed in [152]). L1 also binds in *cis* to FGFR and integrins. Interaction with FGFR (the membrane proximal fibronectin type III (F3) module of L1 presumably interacts with the CAM homology domain, CHD, in the FGFR) occurs as a result of homophilic L1 binding and results in an activation of the FGFR [38]. Association of L1 with integrins modulates cell adhesion to ECM and increases cell motility and migration [41]. L1 presumably interacts both with RGD-binding integrins (through a conserved RGD motif in the Ig6 module of L1 [168]), and with non-RGD-binding integrins (through the third F3 module of L1, [148]). How L1 modulates integrin-mediated adhesion, however, remains unknown.

Intracellularly, a conserved motif in the cytoplasmic domain of L1 interacts with the cytoskeleton-associated proteins including ankyrin, which *via* the cytoskeletal protein spectrin couples to F-actin. When this motif is tyrosine phosphorylated, the microtubule-associated protein doublecortin (DC) is recruited, which in turn can couple with microtubules. These two interactions coupling L1 to the cytoskeleton may play a crucial role in L1 function. Furthermore, L1 binds the adaptor protein AP-2, which is involved in endocytosis [66].

Several models for homo- and heterophilic L1 binding have been proposed involving from one [197] to six [52] Ig-modules of the molecule (reviewed in [53]). A study based on pathological missense mutations of human L1 suggests that the first four domains of L1 adopt a horseshoe structure stabilized by Ig1-Ig4 and Ig2-Ig3 interactions ([4], Fig. (**1C**,**I**). However, this predominant conformation may reversibly open resulting in an extended conformation, in which Ig1-Ig6 assumes a linear, rod-like shape (Fig. (**1C**,**II**)). Correspondingly, two putative mechanisms of homophilic L1 *trans*-interactions exist. In the first model, horseshoe-like structures of opposing L1 molecules do not open up, but rather interact with one another in *trans*. The interaction is mediated by non-covalent bonds between Ig3 of one protein with the horseshoe fold of the other L1 molecule ('cooperative mode', Fig. (**1C,1**)). The second proposed mechanism suggests that Ig1-Ig4 first converts into its extended conformation, and then binds to Ig1-Ig4 of an opposing L1 protein ('modular mode', Fig. (**1C,2**)). Interestingly, with some ligands, the extracellular region operates in a modular mode (binding is mediated by individual Ig domains), whereas with other ligands, it operates in a cooperative mode (several Ig domains form a horseshoe structure that is critical for binding, Fig. (**1C**)). The modular mode of interaction has been demonstrated for such ligands of L1 as neurocan and integrins. The cooperative mode is employed for L1 binding to itself, Ig CAMs, axonin-1, and contactin [53]. In addition, L1 has also been shown to spontaneously trimerize *via* its third F3-module. The resulting L1 trimers have a higher affinity for both homophilic and heterophilic ligands and increase the neurite-promoting ability of L1 in PC12 cells [50].

### NCAM

The neural CAM, NCAM (Fig. (**1A**), reviewed in [177]), belongs to the Ig family of CAMs. As a result of alternative splicing, several NCAM isoforms are generated, in particular the transmembrane molecules of 140 kDa and 180 kDa, which differ in the size of their C- (cytoplasmic) termini, and the 120-kDa isoform attached to the cell membrane by a GPI anchor. The three isoforms show stage- and cell type-related differences in expression. NCAM-180 is predominantly expressed by neurons late in development at postsynaptic densities, NCAM-140 is expressed by both glial cells and neurons localizing to growth cones and axons, whereas expression of NCAM-120 is restricted to glia. Also, the three isoforms induce partially diverging signalling pathways (see Section II for details). NCAM carries an unusual carbohydrate moiety polysialic acid (PSA), posttranslationally attached to the Ig5 module and currently thought to reduce NCAM-mediated adhesion (reviewed in [75]). Besides, sequences encoded by the exons termed VASE, a, b, c, and AAG can be optionally inserted in the extracellular part of NCAM. During development, expression of PSA-NCAM decreases, whereas the amount of VASE-containing transcripts rises. Presumably, these two processes conjointly switch the function of NCAM from a plasticity- to a stability-promoting molecule. The cytoplasmic tail of NCAM-140 and -180 can be phosphorylated at serine and threonine residues [5], and the phosphorylation on at least one threonine is involved in the NCAM-mediated activation of the transcription factor NFκB [98]. Recently, tyrosine phosphorylation of NCAM also was reported and demonstrated to have an inhibitory effect on neurite outgrowth [30].

Heterophilic partners of NCAM include ECM-associated molecules (such as agrin and neurocan, reviewed in [14]), TAG-1/axonin-1 [105], and GDNF and GFRα receptor [115]. The Ig4 module of NCAM also binds to L1 in the *cis*-position. This association seems to be dependent on simultaneous NCAM-NCAM binding, and has a synergistic effect on L1-mediated cellular adhesion, a phenomenon, which has been termed "assisted homophilic L1-L1 *trans*-binding" [64, 65, 86]. In addition, the second F3 (F3,2) module of NCAM interacts in *cis* with the third immunoglobulin (D3) module of FGFR resulting in the induction of intracellular signalling [72]. This interaction has been shown to be involved in NCAM-mediated neurite outgrowth and survival rendering the site of the NCAM-FGFR interaction an attractive target for drug design ([72], see Section IV for details). The first F3 module of NCAM (F3,1) also may interact with FGFR, since antibodies specific for F3,1 inhibit NCAM-stimulated neurite outgrowth [3]. Moreover, peptides derived from NCAM-F3,1 stimulate FGFR activation and FGFR-dependent neurite outgrowth and cell survival (see Section IV for details).

The major cytoplasmic partners of NCAM are the signalling molecules phospholipase Cγ (PLCγ, [38]), protein kinase Cβ (PKCβ, [94, 82]), growth-associated protein 43 (GAP-43, [104]), and the Src family non-receptor tyrosine kinase Fyn [7]. Binding with Fyn is mediated by a direct interaction between NCAM and RPTPα [10]. In addition, NCAM interacts with cytoskeletal proteins. Both NCAM-140 and NCAM-180 can associate with α-and β-tubulin [15], and NCAM-180 is the predominant isoform binding brain spectrin [120], which in turn interacts with multiple targets including filamentous actin, Ca^2+^ pumps, voltage-gated Na^+^ channels, and acidic phospholipids (see [94] and references therein).

Early models of homophilic NCAM interaction suggested either a binding between the Ig3 modules of opposing NCAM molecules or an antiparallel binding between all five Ig domains. However, a recent crystal structure of the Ig1-Ig2-Ig3 triple module of NCAM suggests a more complicated mechanism ([155], Fig. (**1D**)). In this model, the Ig1 and Ig2 modules mediate *cis*-dimerization of NCAM, whereas the Ig3 module mediates *trans*-interactions between NCAM molecules through simultaneous binding to the Ig1 and Ig2 modules (Fig. (**1D**), top). This arrangement results in two perpendicular zippers forming a double zipper-like NCAM adhesion complex (Fig. (**1D**), bottom).

## II. SIGNALLING BY CAMS IN THE NERVOUS SYSTEM

All CAMs have important and varied functions in regulating cell behaviour and tissue organization. In addition to supporting stable cell-cell contacts, they also participate in numerous dynamic events such as neurite outgrowth, cell proliferation, synaptogenesis, and tumour progression (see Section III for details). Such diverse outcomes of CAMs interactions are not surprising, since CAMs not only contribute to cell adhesion, but also act as cell-signalling receptors. The CAMs described here use both 'common' and 'specific' signalling pathways to initiate cellular events. The 'common' signalling cascade can be activated by all three CAM types and is associated with the FGFR, whereas 'specific' signalling cascades are induced through the cytoplasmic tails of the respective CAM molecules.

### CAM Signalling *via* the FGFR

Neurite outgrowth induced by activation of N-cadherin, L1, and NCAM has been shown to require the tyrosine kinase activity of FGFR [185]. This receptor also is involved in CAM-induced survival [151, 108] and regulation of synaptic function [95, 172]. It must be noted, however, that the degree of FGFR involvement in CAM-induced signalling varies depending on the cell type and developmental stage ([140], see also [71] and references therein). FGFR belongs to a family of high affinity receptor tyrosine kinases and is comprised of three Ig modules (D1-3) and a split tyrosine kinase domain. Four FGFR tyrosine kinases (1-4) have been identified so far, the FGFR1 being the most ubiquitous FGFR type in the nervous system. FGFR is thought to be required for CAM-induced neurite extension, since differentiation can be abolished by antibodies against FGFR, by peptides competitively disrupting the FGFR binding to CAMs, by the expression of a dominant-negative FGFR, and by specific FGFR antagonists (see [70] and references therein). The D1-D2 domains of FGFR1 (Fig. (**2A**)) have been shown to associate with N-cadherin (CD4, [188]) and with L1 (presumably the fourth F3 module, [38]), and the D2-D3 of FGFR binds to NCAM (F3 modules, [72]). Extracellular interactions between CAMs and FGFR induce dimerization and autophosphorylation of FGFR (Fig. (**2A**), see [73] for details). Subsequently, the cytoplasmic tail of FGFR serves as a docking site for several proteins. One of them is PLCγ, which becomes activated upon binding and cleaves phosphatidylinositolbisphosphate (PIP2) to generate inositol-trisphosphate (IP3) and diacylglycerol (DAG). IP3 induces Ca^2+^ release from intracellular Ca^2+^ stores, whereas DAG can be converted to 2-arachi-donoylglycerol (2-AG) and arachidonic acid (AA), which both can induce Ca^2+^ entry *via* plasma membrane Ca^2+^ channels ([71] and references therein). The resulting increase in the cytoplasmic Ca^2+^ concentration leads to the activation of calmodulin kinase II (CaMKII), and, subsequently, the cAMP response element binding protein, CREB. Moreover, Ca^2+^ and/or DAG stimulate PKC, which in turn launches the signalling cascade Raf-Ras-MEK-MAPK also resulting in the activation of CREB [81]. FGFR signals are also transduced *via* the FRS2/FRS3-SHP2-GRB2 docking protein complex to the SOS-Ras-MAPK and GAB1/GAB2-PI3K-PDK-Akt signalling cascades. Phosphatidylinositol 3-kinase (PI3K) and Akt are intimately involved in the CAM-mediated neuronal survival [99, 108, 31]; moreover the PI3K activation leads to the phosphorylation of Akt [156]. The targets of Akt include the transcription factors CREB and NFκB [40, 67]; however, the genes activated by NFκB or CREB downstream of CAM and responsible for CAM cellular effects remain to be identified.

### Signalling Pathways Associated with Cytoplasmic Domains of CAMs

#### Cadherins

The cytoplasmic tail of classic cadherins binds to various cytosolic and membrane proteins (Fig. (**2B**), left), including catenins, kinases, phosphatases, heterotrimeric G proteins, adaptor proteins, and presenilin 1 (see [28] and references therein). It contains two major binding regions: the C-terminal domain and the juxtamembrane domain (JMD, situated proximal to the plasma membrane). The C-terminal domain mainly binds to γ-catenin and the β/α-catenin complex, which anchors cadherins to the actin cytoskeleton [79]. The JMD interacts with p120-catenin family members and participates in the regulation of cell-cell adhesion and cell physiology by affecting cadherin clustering, cytoskeletal dynamics and calcium influx (reviewed in [1]). In addition, p120-catenin interacts with nonreceptor tyrosine kinase Fyn, which is involved in multiple intracellular signalling events (see below).

In addition to their role within the cadherin complex, both the β- and p120-catenin subfamilies contribute to signalling when uncoupled from cadherins, in particular by regulating Rho GTPase activity [2, 111]. Small GTPases of the Rho family (Rac1, Cdc42, and RhoA) are modulated upon homophilic binding of cadherins, and, moreover, they can reciprocally affect cadherin functions [194]. The effect of cadherins on each Rho GTPase varies depending on the cellular context and cross-talk between different Rho-GTPases; however, the most well characterized responses triggered by cadherin ligation are the activation of Rac1 and the inhibition of RhoA (Fig. (**2B**), see [194, 184] and references therein), which occur, respectively, within minutes and hours of cadherin binding. Activation of Rac1 regulates actin polymerization by recruiting the actin nucleator complex Arp2/3 to cell contacts thereby stabilizing the cadherin complex (see [84] for details).

There has not been established an enzymatic link between cadherin ligation and the inhibition of RhoA. However, p120-catenin not bound to cadherin interacts with RhoA [101] and inhibits its activation [2]. These activities of p120-catenin are dependent on its interaction with the JMD [111, 118]. Thus, p120-catenin may exist in two distinct states in the cytoplasm: (1) cadherin-bound, not affecting Rho GTPase activity, and (2) unbound, interacting with RhoA and preventing its activation (Fig. (**2B**)). The switch between these two states would provide a mechanism for regulation of cytoskeleton dynamics and Ca^2+^ concentration in the vicinity of the junction. Interestingly, the clustering of N-cadherin observed during synaptic maturation *in vivo* is associated with the uncoupling of p120-catenin [134], and cytosolic pools of p120-catenin can be detected in the developing CNS [22]. It has been suggested that at immature synaptic contacts, N-cadherin is present in small clusters with restricted adhesive activity, which include bound p120-catenin. In this case, Rac1-associated signalling is activated contributing to the organizing of the actin cytoskeleton and to the assembly of the synaptic junction. During synaptic maturation, N-cadherin clusters into larger strongly adhesive puncta as a result of cadherin homophilic binding and/or synaptic activity. As a result, p120-catenin uncouples from the N-cadherin complex and inactivates RhoA leading to disassembly of contractile actin-myosin filaments. This process contributes to the stabilization of the synaptic complex and to the increase in synaptic efficacy. In addition, inhibition of RhoA may induce Ca^2+^ entry into the cytoplasm by activating voltage-gated Ca^2+^ channels of the plasma membrane further increasing synaptic function [118].

The signalling pathways associated with the cytoplasmic domains of protocadherins remain elusive (Fig. (**2B**), right). Protocadherin 18 supposedly tyrosine phosphorylates the docking protein Dab1 upon binding. One important consequence of Dab1 tyrosine phosphorylation might be the nucleation of the multi-protein signalling complex Fes which modulates cytoskeletal dynamics (see [158] for review). Phosphorylated serine/threonine phosphatase type 1α (PP1α) activated by the protocadherin 7 might modulate the activity of caspase-9 involved in neuronal apoptosis [29] and IP3-induced Ca^2+^ release from intracellular stores [165]; however, no direct relationship between protocadherin ligation and these events has so far been demonstrated. The most well-established signalling pathway associated with the cytoplasmic tail of protocadherins involves the tyrosine kinase Fyn, which serves as a docking protein for the focal adhesion kinase, FAK. FAK interacts with numerous proteins and may activate the MAPK pathway *via* several routes [139]. In particular, recruitment of FAK leads to a sequential activation of the small GTP-binding protein c-Ras1 and the serine/threonine kinases c-Raf-1 and MAPK1/2. The phosphorylation of MAPK1/2 eventuates in the activation of the transcription factors CREB and Fos [27]. Interestingly, Fyn is also associated with the cadherin complex *via* p120-catenin and phosphorylates β-catenin on Tyr142, the residue essential for the α-catenin-β-catenin interaction [119]. Thus, Fyn may reciprocally modulate the cadherin complex. In addition, Src family kinases including Fyn can positively regulate non-selective cation channels of the plasma membrane [54, 175] and L-type voltage-gated Ca^2+^ channels ([59], and references therein), thus increasing Ca^2+^ entry into the cytoplasm.

#### L1-CAM

Like cadherins, L1 and NCAM also activate the MAPK signalling pathway. In **L1**-induced signalling, activation of the nonreceptor tyrosine kinase p60c-src (Src) is required for MAPK phosphorylation (Fig. (**2C**, [141]). Cross-linking of L1 molecules on the growth cone membrane can induce internalization of L1 *via* Src. This internalization is mediated by the small GTPase dynamin and leads to a sequential activation of PI3K, Rac, and MAPK (Fig. (**2C**), [141]). The L1-mediated MAPK activation also induces the expression of several genes associated with motility and invasion [149]. MAPK2 in turn can phosphorylate L1 at serines; thus, L1 signalling may reciprocally regulate L1 function ([138], Fig. (**2C**)).

Integrins as binding partners of L1 can also contribute to the L1-induced signalling. In particular, β1 integrins also signal *via* Src, PI3K, and Rac1 to activate MAPK [126] raising the possibility that L1 and integrins may cooperate on some intracellular signalling 'routes' and/or signal *via* each other.

#### NCAM

For NCAM, phosphorylation of MAPK1/2 was demonstrated in primary neurons following NCAM clustering on the cell surface, homophilic *trans*-binding, and treatment with synthetic peptide ligands (see [70] for references). Clustering of NCAM-140, but not NCAM-180, resulted in MAPK phosphorylation, and this event was mediated by activation of the Fyn-associated signalling cascade (Fig. (**2D**), [140]). Interestingly, Fyn was recently co-precipitated also with NCAM-180 [10] and shown to interact with both NCAM-140 and -180 *via* receptor protein tyrosine phosphatase α (RPTPα). The affinity of the RPTPα-NCAM-180 interaction was lower than that of the RPTPα-NCAM-140 interaction, but these observations suggest that an NCAM-180-Fyn interaction might also occur.

Association between NCAM and Fyn has been shown to be 'reinforced' by the cytoskeletal protein spectrin, which predominantly binds to NCAM-180, but also to NCAM-140 [160], and serves as a scaffold for the NCAM-RPTPα-Fyn complex. Another important function of spectrin may be recruitment of the growth-associated protein GAP-43. GAP-43 presumably coexists in complexes with NCAM at the plasma membrane and is known to be indispensable for the NCAM-induced neurite outgrowth (Fig. (**2D**), see [83] and references therein). Moreover, recent experimental evidence suggests that expression of GAP-43 might act as a 'switch' between NCAM-140- and NCAM-180-induced signalling [83]. In the presence of GAP-43, NCAM-induced neurite outgrowth requires functional association of NCAM-180/spectrin/GAP-43, whereas in the absence of GAP-43, the NCAM-140/Fyn-associated signalling pathway seems pivotal [83].

To participate in signalling impinging on neurite outgrowth, GAP-43 has to be phosphorylated and thereby activated by protein kinase C (PKC, [83] and references therein). PKC is activated as a result of triggering the FGFR-associated pathway, and also contributes to the activation of the Fyn-cascade joining it at the level of Raf (Fig. (**2D**), [81]). NCAM might also activate heterotrimeric G-proteins [36] resulting in stimulation of adenylyl cyclase and production cAMP. This leads to the activation of PKA and its effectors CREB and another transcription factor, c-fos (Fig. (**2D**), [62]). Finally, NCAM can *via* nitric oxide synthase (NOS) induce production of cyclic guanosine monophosphate (cGMP), which in turn activates protein kinase G. This signalling pathway is involved in NCAM-induced neurite outgrowth and survival in primary neurons [32].

## III. PHARMACOLOGICAL TOOLS TO STUDY CAM FUNCTION

The first pharmacological tools developed to study CAM functions were antibodies targeting specific CAM epitopes followed by CAM-derived recombinant proteins. In this section, we describe the advantages of these approaches *in vitro* and *in vivo* as well as their drawbacks, which have been partially compensated for by introduction of structure-based design of CAM ligands (discussed in Section IV). The major pharmacological approaches used for studying CAMs are summarized in Table **1**.

### Cadherins

Cadherins have important and varied functions in the regulation of cell behaviour and tissue organization. Besides supporting stable cell-cell contacts, they also participate in dynamic events. In the nervous system, these include cell sorting and tissue segregation [49], cell locomotion during gastrulation [13], neurite growth and guidance [103, 127], synaptogenesis and modulation of synaptic function ([11, 60, 164], reviewed in [163]). Traditionally, cadherin function has been studied by monitoring the behaviour of cultured cells as they grow to confluence and form adhesive contacts [13, 92]. However, this method has significantlimitations because (1) homophilic cadherin ligation is difficult to evaluate, and (2) the time-course of adhesion formation does not allow to study cadherin effects onrapidly changing cellular parameters (e.g., the levels of enzyme activities or the intracellular Ca^2+^ concentration). Another common approach, the 'Ca^2+^ switch method', exploits the Ca^2+^ dependency of homophilic cadherin adhesion. In this approach, the formation of cadherin-mediated junctions is manipulated by varying the level of extracellular Ca^2+^. At low extracellular Ca^2+^, cadherin function is blocked; restoration of the Ca^2+^ level results in the reformation of cell junctions and induction of cadherin-mediated intracellular signalling [112, 21]. These two methods have successfully been used to 'switch on' the cadherin-mediated signalling, whereas 'switching off' mostly was accomplished by using inhibitory antibodies to cadherins. In combination, these approaches were used to study the regulation of the function of C-cadherins during activin-induced morphogenesis [13], the modulation of Rho GTPases and β-catenin by cadherin ligation [21], the role of N-cadherin in the growth and targeting of thalamocortical axons [121] and in the formation of Schwann cell junctions [181], the role of Src kinases in the stabilization of cadherin-mediated adhesion [114], the involvement of R- and B- cadherins in the axonal guidance of the optic nerve [48], the role of cadherins in the subcellular organization of the postsynaptic density [56], the modulation of long-term potentiation by a protocadherin Arcadlin [191], and for the identification of an N-cadherin motif interacting with FGFR [185, 188].

However, the major drawback of the above approaches is that they are not able to distinguish between the direct cadherin signals (those activated as direct consequences of cadherin ligation) and the juxtacrine signals (those requiring cadherin adhesion to bring cell surfaces together, but not being direct consequences of cadherin ligation). This distinction is fundamental for any rigorous analysis of cadherin function.

An important advance was the development of recombinant cadherin-specific ligands. Several proteins have been produced encompassing the complete ectodomains of C-cadherin [13], N-cadherin [173, 91], or E-cadherin [85, 110]. When presented on planar substrata or coated on beads, these ligands supported cadherin-specific adhesion [13], lateral clustering [193], and neurite outgrowth [173] recruiting catenins and regulating the cytoskeleton [85, 91]. These proteins have proved to be powerful tools to separate the cadherin-activated from cadherin-dependent cell signals, in particular to evaluate the time-course of cadherin-induced modulation of Rac and Rho [112, 21]. They have also helped establishing a novel method for studying the dynamics of functional anchoring of cadherins to the actin cytoskeleton. In this method, a biophysical approach was used enabling the authors to monitor the two-dimensional movement of single or small clusters of proteins [90]. Ligand-coated particles were forced to contact different domains of the plasma membrane using optical tweezers. Thereafter, the movement of the particles bound to membrane receptors could be monitored with nanometer precision by video microscopy and single-particle tracking [157]. Using cadherin-coated beads as ligands, it was thus possible to follow the anchoring of E-cadherins [137] or N-cadherins [91, 170] to the cytoskeleton, and to perform dose-response analysis and pharmacological perturbations to study the molecular mechanisms controlling the anchoring.

Also, a recombinant ligand corresponding to the intracellular regions of cadherins was generated. The juxtamembrane domain (JMD) of cytoplasmic tail of N-cadherin was produced and used to study the functional link between the N-cadherin JMD and voltage-gated Ca^2+^ channels. It was found that the infusion of the soluble JMD into ciliary neurons decreased the amplitude of the high-threshold voltage-activated Ca^2+^ current, and this effect was mediated by RhoA [118].

### L1-CAM

The L1-CAM plays a pivotal role in the function of the nervous system (reviewed in [58]; see also [39] and references therein). During development, it participates in regulation of neuronal migration and survival, neurite outgrowth, axonal pathfinding, and synaptogenesis. In the adult, L1 is involved in synaptic plasticity, including spatial learning and memory and induction and maintenance of LTP in the hippocampus. Mutations of L1 in mice and humans have been demonstrated in X-linked chromosomal diseases, such as the MASA syndrome (mental retardation, aphasia, shuffling gait), spastic paraplegia type 1, and agenesis of the corpus callosum [26, 69]. This multiplicity of L1 functions can be explained by its ability to bind numerous cell surface and ECM molecules through different segments of its ectodomain. However, early studies on L1 function did not attempt to segregate activities associated with individual L1 modules. Purified L1 and/or antibodies were mostly utilized to investigate the cellular effects of L1 and their molecular mechanisms. First, inhibitory Fab antibodies to L1 were produced, which modified the migration of external granular cells from mouse cerebellum [97]. These Fab fragments have also been shown to decrease the calcium-independent aggregation of mouse cerebellar cells and neuroblastoma N2A cells [125]. Other monoclonal antibodies to L1 have been demonstrated to suppress the binding between L1 and axonin-1 [87]. To assess the binding in this study, purified L1 and axonin-1 were covalently coupled to the fluorescent microspheres, and their aggregation was analyzed by flow cytometry. Inhibitory antibodies were also used to investigate the cell type specificity of L1-mediated cell adhesion [68], the role of L1 in neurite outgrowth/fasciculation [43], in TAG-1 induced neurite extension [42], and in Schwann cell differentiation [145]. Moreover, inhibitory antibodies to specific regions of L1 have been generated. In particular, monoclonal antibodies to an oligomannosidic carbohydrate of L1 have been shown to inhibit complex formation between L1 and NCAM [57], suggesting that the carbohydrate moiety mediates the L1-NCAM binding. More recently, activating antibodies were produced capable of triggering L1-mediated signalling, most probably by clustering L1 molecules on the cell surface [78, 141, 39]. These antibodies have been used to elucidate the effect of L1 stimulation on the activity of phosphatases in the growth cone-enriched membranes [78], to study the involvement of the MAP kinase pathway in L1-induced signalling [141], and to demonstrate that ethanol disrupts the signalling link between L1 clustering and MAPK activation [166]. The antibodies enhanced neurite outgrowth from several populations of neurons and promoted neuronal survival *in vitro* [39], thus mimicking some beneficial functions of homophilic L1 binding *in vitro* and suggesting that they may serve to trigger these functions *in vivo*.

The toolkit for studying L1 signalling has been vastly extended by the advent of recombinant L1 proteins. Doherty *et al.* [37] have demonstrated that a soluble recombinant L1-Fc chimera stimulates neurite outgrowth from primary neurons in a manner similar to that of native L1, and that neuronal FGFR is required for this response. L1-Fc and L1 purified from mouse brain have been used to investigate the signalling pathways involved in L1-triggered neuroprotection [99]. Furthermore, from recent studies using an array of recombinant L1-derived proteins, a theory has emerged suggesting that L1 can interact with its ligands in both a modular and a cooperative modes (see Section I for details). The 'modular model' was supported by studies showing that some binding activities of L1 were restricted to individual domains. In particular, it was found that the Ig1 module supports L1 interaction with the cell surface proteoglycans neurocan, whereas RGD sites in the Ig6 domain are indispensable for L1- integrin binding [113]. More recently, another site in L1 involved in the L1-integrin interaction was identified using a range of recombinant L1-proteins [148]. To investigate the function of F3 domains of L1, the authors produced a series of constructs including two or three adjacent F3 modules (F3,1-2; F3,2-3; F3,1-3), the Ig6 module alone, or Ig6 in conjunction with adjacent F3 modules (Ig6-F3,1-2 and Ig6-F3,1-3), as well as the whole L1 ectodomain. Using adhesion and immunoprecipitation assays, it was shown that the third F3 module of L1 spontaneously forms homotrimers and higher order complexes, and these complexes support direct RGD-independent interactions with severalintegrins.

A modular mode of interaction has also been demonstrated for L1 binding with neuropilin-1 (NP-1). L1 is known to associate with NP-1 and is required as part of the NP-1/Semaphorin 3A (Sema 3A) receptor complex for axon guidance responses [18]. Using L1 constructs containing missense pathological mutations, Castellani and coworkers [19] have shown that these responses are initiated by a specific *trans* binding of L1 to NP-1, and have confined the L1-NP-1-binding site to the Ig1 module of L1. A peptide derived from this region could reverse the axonal guidance effects of Sema3A, and a missense mutation located in this sequence specifically disrupted both L1-NP-1 complex formation and Sema3A signalling. Finally, Zhao and Siu [197] have mapped the homophilic binding domain in L1. Several recombinant proteins (Ig1, Ig1-2, Ig1-2-3, Ig4-5-6, and fibronectin modules) were prepared and coupled to covaspheres, and their homophilic binding activity was determined using a covasphere-to-substratum binding assay. Only the Ig2, Ig1-2, and Ig1-2-3 constructs were capable of homophilic binding and of inducing neurite extension, and the Ig2-Ig2 interaction was inhibited by anti-Ig1-2-3 antibodies and soluble Ig2. Based on these results, the authors proposed that Ig2 was necessary and sufficient for L1 homophilic *trans*-interaction.

However, subsequent studies of the mechanism of homophilic L1 adhesion have expanded this view. In particular, EM visualization and sedimentation analysis of the Ig1-Ig6 domains of L1 revealed that the protein exists in two conformations: an extended molecule and a globular structure with a linear tail, where Ig1-Ig4 engaged in a horseshoe fold, and the tail represented Ig5-Ig6 [142]. These data suggest that with some ligands L1 operates in a cooperative mode, where the horseshoe structure formed by the Ig1-Ig4 domains is critical for the L1-ligand interaction. This hypothesis has been corroborated by the studies of De Angelis *et al.* [4], where a number of L1-Fc fusion proteins were generated with missense mutations that are associated with disease. It was found that mutations located throughout Ig1-Ig6 and F3,2 interfered with both homo- and heterophilic interactions. More recently, the recombinant L1-Fc proteins expressed in eukaryotic cells were used to identify a minimal segment in the L1 ectodomain mediating homophilic interaction [52]. The results indicate that Ig1-4 of L1 are critical for homophilic binding and neurite outgrowth; however, a larger portion of the protein, comprising Ig1-Ig6, is required for optimal promotion of cell adhesion and neurite outgrowth.

Interestingly, the pharmacological use of L1-derived recombinant proteins *in vivo* has also been attempted. A soluble L1-Fc construct promoted locomotor recovery in rats after spinal cord injury [133]. Also, L1-Fc accelerated rat optic nerve regeneration when presented on the surface of the nerve [190]. However, therapeutic application of recombinant L1-proteins might be limited because of difficulty in obtaining these reagents under highly reproducible conditions and/or in large amounts.

### NCAM

NCAM is involved in numerous physiological and pathological processes (reviewed in [177]). These include cell proliferation [106] and motility [122], neuronal differentiation [109] and survival [31], axon guidance [179], synaptic plasticity [192], and learning and memory formation [100]. Some of the NCAM effects are regulated by the expression of PSA (see [177]). Genetic deletion of NCAM does not result in severe brain dysfunction. However, NCAM-deficient mice have a decreased brain weight and demonstrate significant alterations in the olfactory system and hippocampus. They also exhibit defects in spatial learning and an anxiety-like behaviour. Re-expression of NCAM-180 in these mice can compensate for some of the behavioural alterations caused by the NCAM knockout [159]. Interestingly, simultaneous deletion of the genes responsible for NCAM polysialylation results in a severe phenotype including postnatal growth retardation and early death [183] indicating an important role for PSA in controlling NCAM interactions during development.

As well as for cadherins and L1, initial studies on the NCAM function were carried out mostly using NCAM-specific antibodies. NCAM was first described by Jorgensen and Bock [63] as a brain specific antigen termed D2; however, the functional role of D2 as a cell adhesion molecule had not been ascertained. Thiery *et al.* [169] have independently identified NCAM as one of the molecules responsible for the *in vitro *adhesion of chick retinal cells and prepared anti-NCAM antibodies capable of inhibiting cell adhesion. These antibodies have been used to demonstrate the existence of Ca^2+^-dependent (cadherins) versus Ca^2+^-independent (L1- and NCAM-mediated) adhesion systems [12]. Data on aggregation of NCAM-containing lipid vesicles and on NCAM binding to cells coated with anti-NCAM Fab fragments strongly suggested that NCAM molecules on different cells may interact directly to mediate cell adhesion [135]. Further studies using antibodies to NCAM have shown that its function is required for neurite growth, guidance and fasciculation (reviewed in [136]), and interactions between spinal cord neurons and muscle cells ([24] and references therein).

At the same time, the existence of structurally and functionally different regions in the NCAM ectodomain was established [24]. To isolate NCAM regions containing a homophilic binding site or PSA moieties, the authors have used temperature- and enzyme-induced generation of NCAM fragments as well as NCAM antibodies ([24] and references therein). Watanabe *et al.* [182] performed a systematic study mapping structural and functional determinants of NCAM. Twelve different monoclonal antibodies were produced, each recognizing a distinct NCAM epitope. Some of the antibodies were inhibitory, whereas others enhanced the NCAM-mediated cell adhesion. The authors also generated a set of peptide fragments from NCAM and used their size and reactivity to construct an epitope map of NCAM [47]. As a result, the location of functionally important sites of the NCAM molecule has been refined.

More recently, NCAM antibodies have been employed to study the signaling pathways initiated by NCAM-140 [140] and the role of GAP-43 in NCAM-induced neurite outgrowth [104]. Also, antibodies to NCAM have been widely used to study the physiological role of the molecule *in vivo*. It has been found that NCAM is involved in the migration of luteinizing hormone-releasing neurons into the mouse brain [143], in the initial nerve ingrowth and ramification in the chick muscle [93], in the limb regeneration in newts [102], and in the long-term potentiation in rats [100, 128].

The advance in the studies of NCAM function has been vastly accelerated by demonstrating that its ectodomain includes five contiguous segments belonging to the immunoglobulin superfamily [25]. These authors also were first to suggest that interactions between Ig modules could be the basis for NCAM homophilic binding, and using NCAM fragments they identified Ig1, Ig2, and Ig3 as important for this binding. More recently, Rao *et al.* [124] combined three approaches to identify NCAM regions involved in homophilic interactions: (1) analysis of domain deletion mutations, (2) mapping of epitopes of monoclonal antibodies, and (3) use of synthetic peptides to inhibit NCAM function. An attempt has also been made to evaluate a role of individual modules of NCAM in different cell activities [46]. The authors produced Ig1 to Ig5, as well as the first and second F3 modules, and investigated their effects on cell adhesion, neurite outgrowth, fasciculation of neurites, and orientation of inhibitory interneurons in the cerebellar cortex. Interestingly, no role for Ig3 in cell adhesion was found in this study. Nevertheless, the ability of recombinant Ig3 to self-aggregate has subsequently been shown [123] confirming that this domain might play an important role in NCAM-mediated cell adhesion.

An interesting strategy to study the role of L1 and NCAM in neurite outgrowth has been used by Takei *et al.* [161], who have employed a microscale chromophore-assisted laser inactivation (micro-CALI) assay to perturb the function of these proteins at precise times in single growth cones of embryonic dorsal root ganglion neurons. CALI induces loss of function of specific proteins in living cells by using antibodies to these proteins conjugated with dye malachite green. The dye absorbs 620 nm laser light and generates short-lived hydroxyl radicals that inactivate proteins within a small radius around the irradiation site (reviewed in [180]). Since inactivation only occurs when irradiation is initiated, it is possible to determine the time-course of cellular effects induced by such a 'local knockout' of a given protein. Using this method, the authors found that inactivation of NCAM had drastically different effects on growth cone protrusion and neurite extension as compared to L1 suggesting distinct roles of these proteins in different stages of neurite outgrowth.

An important target in NCAM pharmacology is the specific carbohydrate, polysialic acid (PSA), posttranslationally attached to the Ig5 module of NCAM. NCAM is the predominant carrier of PSA, and expression of PSA-NCAM is associated with developmental and learning-related neural plasticity (see Section I for details). Therefore, modulation of PSA levels may be a promising pharmacotherapy for treating a wide range of neurological disorders.

The main analytical approach to study the role of PSA-NCAM has thus far been the use of an enzyme endoneuraminidase (Endo-N) to remove PSA moiety from NCAM. PSA contains up to 200 residues of sialic acid linearly linked by an unusual α2,8-bond, and Endo-N is specific for α2,8-linked sialic acid polymers with a minimum chain length of five [176]. Therefore, the enzyme does not degrade any other known sialic acid-containing structures and is thus a convenient tool to study the function of PSA-NCAM. The enzymatic approach has been used in a number of studies to demonstrate the role of PSA-NCAM in axonal growth and branching [34, 196], cell migration [74, 6], and synaptic plasticity [107, 33]. However, this strategy only allows one to address the effect of abrogation of PSA function. Recently, Vaithianathan *et al.* [174] have used a bacterially derived PSA, colominic acid, to mimic PSA effects in primary neurons. This technique allowed the authors to study the direct modulation of neuronal receptors by PSA.

Other promising candidates for manipulating PSA-NCAM are synthetic peptides that can mimic PSA functions. Recently, using a monoclonal antibody specific for PSA, a phage-display library was screened leading to the identification of two cyclic nonapeptides that are PSA epitope analogues. *In vitro*, the peptides promote axon growth, defasciculation and migration of neural progenitors [171]. *In vivo*, the peptides enhance migration of grafted neuroblasts in mouse brain and modify the trajectory of retinal ganglion cell axons in developing chicken retina [171]. The PSA mimetic peptide pr2 improves spatial long-term performance in mice following post-training intra-hippocampal injection [44]. Thus, peptides capable of enhancing biological activity of PSA are not only good tools for 'switching on' PSA function, but may also have a therapeutic potential.

## IV. STRUCTURE-BASED DESIGN OF CAM MIMETICS

Significant progress in the design of CAM-specific ligands was achieved due to the solution of the 3D structure of various CAMs and their fragments. Sites involved in homophilic and/or heterophilic CAM interactions were identified and, when presented as peptides, often proved to have significant effects on CAM function.

### Cadherins

Several cadherin-specific ligands were designed based on the 3D crystal structure of CD1 modules of N-cadherin (see [146] for review). It was demonstrated that CD1 of N-cadherin forms two dimeric structures with well-defined interactions mediating the formation of *cis *and *trans* dimers [147]. Interestingly, the HAV motif at position 79–81, which was previously identified in peptide competition studies as being important for cadherin function [9,35] was implicated in both structures. In accordance with this, Williams and coworkers [186] demonstrated that the short cyclic HAV peptides inhibited N-cadherinfunction, and that the nature of the amino acids that flankedthe HAV motif determined the specificity of thepeptides. For example, when the HAV motif was flanked by a singleaspartic acid, which mimics the natural HAVD sequence of N-cadherin,the peptide became a selective inhibitor of N-cadherinfunction. In contrast, when the HAV motif was flanked by a singleserine, which mimics the natural HAVS sequence of E-cadherin,it lost its ability to inhibit the N-cadherin response. Thus, subtle changes in the amino acids flankingthe HAV motif could account for cadherin specificity. More recently, the same authors have determined amino acid residues in CD1 of N-cadherin (INP) interacting with unique sequences that flank the HAV motif in N-cadherin, and designed a linear peptide mimetic (INPISGQ), which functioned as a highly specific and potent antagonist of N-cadherin function (IC_50_~15 μM, [187]). Peptide mimetics of the corresponding motif in E- or P-cadherin failed to inhibit N-cadherin function. A short cyclic peptide that contained only the INP motif from N-cadherin was also a potent N-cadherin antagonist.

Monomeric versions of the HAVDI and INPISGQ peptides have been used in many studies as specific N-cadherin blockers. However, when cyclic peptides were generated containing a tandem repeat of the individual motifs, they functioned as N-cadherin agonists [189]. Interestingly, the linear dimeric versions of the motifs also stimulated neurite outgrowth, which was inhibited by monomeric versions of the same motif, as well as by recombinant CD1 module of N-cadherin and by blocking of FGFR-associated signalling. These data suggest that the peptides function by binding to and clustering N-cadherin in neurons and thereby activating an N-cadherin/FGFR signalling cascade. Moreover, the agonists also promoted the survival of several populations of CNS neurons in an FGFR-dependent manner [151]. Thus, the cadherin-derived peptides not only represent promising tools for the study of the cadherin-mediated signalling, but may also be used for developing therapeutic agents that promote cell survival and axonal regeneration.

### L1-CAM

The ligands derived from the 'active' sites of the L1-interacting molecules and L1 itself may as well be good candidates for analytical and therapeutic use. However, the structure of the L1 ectodomain at the atomic resolution has not yet been solved. Recently, crystallization and preliminary X-ray analysis of the extracellular Ig1-4 and F3,1-3 of L1 were attempted. Although the crystals of the F3,1-3 module of L1 diffracted to a resolution of 2.8Å, it was not possible to solve the structure [88]. The absence of structural details of folding and interactions between L1 modules is a major impediment in designing small molecules binding L1 and mimicking its functions. Horstkorte *et al.* [57] generated a peptide representing a sequence in the Ig4 domain of NCAM, responsible for the NCAM-L1 interaction. This peptide interfered with the NCAM-L1 binding and inhibited neurite outgrowth *in vitro*. Another L1-derived peptide, L1-A, spanning the H178 and G191 residues, has been shown to inhibit L1-mediated homophilic binding. Thus, the sequence HIKQDERVTMGQNG of Ig2 may constitute a part of the L1 homophilic binding site [198]. Williams *et al.* [185] identified a CAM homology domain in the FGF receptor family and showed that synthetic peptides derived from this domain specifically inhibit neurite outgrowth stimulated by L1, N-CAM, and N-cadherin. Conversely, a peptide region in the third F3 module of L1, SVILSGLRPYSSYHL, derived based on its homology to the CAM-homology domain (CHD) of the FGFR, inhibited the L1-induced cellular responses and was thus suggested to represent the FGFR binding site in L1 (see [38] and references therein).

### NCAM

For NCAM as well as for L1, the simplistic modular model of homophilic binding has been replaced by a more complex cooperative model. The X-ray structure of the Ig1-Ig2-Ig3 dimer (Fig. (**2D**), [154]) demonstrated that the cellular functions of NCAM can not unequivocally be assigned to individual modules, because multiple *cis* and *trans* interdomain interactions are involved in homophilic binding. It has also become clear that small molecules (e.g. peptides) able to specifically affect these interactions might be both useful tools for dissecting NCAM-mediated signalling and promising candidates for drug design.

In our research group we have used two strategies to develop pharmacological compounds affecting NCAM function: (1) screening of combinatorial peptide libraries with fragments of NCAM, and (2) structural determination of binding sites in NCAM by nuclear magnetic resonance (NMR) and X-ray crystallography. As a result, a number of small molecular weight agonists and antagonists of NCAM have been developed mimicking or specifically inhibiting NCAM-mediated cellular effects (see [8] for review). Here, we will focus on three of them, the C3, P2, and FGL peptides, since these NCAM mimetics have been extensively characterized *in vitro* and shown to exert prominent effects *in vivo*.

The C3 peptide, ASKKPKRNIKA, was identified by screening a combinatorial peptide library with biotinylated recombinant NCAM Ig1 [129]. To increase the potency of the peptide, C3 was subsequently synthesized as a dendrimer comprising four monomers coupled to a lysine backbone (C3d). C3d was demonstrated to interact with NCAM Ig1 at a site different from that involved in the binding of Ig1 to Ig2 [129]. However, the cellular effects/signalling pathways activated by C3d have been shown to be identical to those activated by homophilic NCAM binding. In particular, C3d induces neurite outgrowth from primary neurons by increasing intracellular Ca^2+^ and activating of FGFR, the MAP-kinase pathway and CREB [130, 131, 62, 154]. The peptide also promotes neuronal survival through phosphorylation of Akt and PKB [31] and interferes with NCAM-mediated cell adhesion presumably by competing with homophilic, physiological NCAM binding [129]. Recently, C3d was demonstrated to inhibit apoptosis induced by interleukin-1β (IL1-β) in beta cells by inhibiting the IL-1β-induced MAPK activity [117].

*In vivo*, the C3d exerts a neuroprotective effect against teratogen-induced developmental defects when administered at embryonic day 14 in rats [76]. However, in adult animals, C3d impairs short- and long-term memory [45, 51, 16] suggesting that in the mature brain, the peptide might disrupt already established neuronal connections. Thus, the C3d peptide appears to differentially affect the developing and the adult nervous system.

The P2 peptide, GRILARGEINFK, is a conserved sequence fragment derived from the Ig2 module of NCAM. Based on NMR and crystallography studies, this peptide has been identified as a contact area in Ig2 interacting with Ig1 [61, 154]. The dendrimeric form of P2 (P2d) binds to recombinant NCAM Ig1 and triggers signal transduction pathways similar to those activated by NCAM. The peptide disrupts NCAM-mediated cell adhesion, induces neurite outgrowth from primary neurons [154], has neuroprotective effect [116], and increases intracellular Ca^2+^ concentration ([Ca^2+^]_i_, [71]). These effects have been shown to be dependent on the intracellular messengers also involved in the NCAM-induced signalling: FGFR, PLCγ, DAG, Akt, and MAPK thus rendering the peptide a convenient tool to mimic 'native' NCAM signalling.

Thus, we recently employed P2 to determine [Ca^2+^]_i_-regulating mechanisms involved in NCAM-induced intracellular signalling, and their importance for NCAM-induced neurite outgrowth [71].Using P2 as an NCAM ligand allowed us to monitor rapid NCAM induced Ca^2+^-responses. This resulted in identifying previously unanticipated mechanisms of Ca^2+^ homeostasis activated by NCAM and contributing to neuronal differentiation.

Other less well characterized small-weight ligands of NCAM include the peptides termed NBP10, P1B, and HBP. The NBP10 peptide has been identified by screening a combinatorial synthetic peptide library with NCAM purified from postnatal day 10 rat brains [132]. This peptide binds NCAM Ig1 as demonstrated by NMR, and modulates cell aggregation and neurite outgrowth, induced by NCAM homophilic binding. The neuritogenic effect of NBP10 is associated with the induction of Ca^2+^ influx from the extracellular space [132]. The P1B peptide is a conserved sequence fragment of NCAM derived from the Ig1 module on the basis of structural studies [155]. This peptide presumably represents the Ig1 contact area in an NCAM *cis* dimer and modulates physiological NCAM homophilic *trans* interaction in a dose-dependent manner [96]. The HBP peptide represents a continuous part of the heparin binding sequence in NCAM Ig2 module. The heparin-binding motif (HBM) in Ig2 has recently been shown to partially overlap with the Ig1-Ig2 contact site [89]. Thus, the peptide derived from the HBM site can be expected to affect/mimic cellular effects of NCAM dependent on the Ig1-Ig2 interaction. Accordingly, the HBP peptide promotes neurite outgrowth from primary neurons.

The *in vitro* effects of the aforementioned peptides suggest that they may have therapeutic applications; however, their *in vivo* effects are awaiting characterization.

The FGL peptide, EVYVVAENQQGKSKA, has been identified as part of the NCAM binding site for the D2-D3 region of the FGFR ectodomain [72]. *In vitro*, FGL binds to and phosphorylates FGFR [72] and increases neurite outgrowth and survival in primary neurons [72, 108].The effects of the FGL peptide depend on activation of FGFR and the MAPK and PI3K intracellular signalling pathways, which also are triggered by the NCAM ligation to FGFR. Thus, FGL mimics NCAM heterophilic binding to FGFR and can be considered as an NCAM-derived agonist of FGFR. This suggests wide opportunities for the peptide to be used both as a tool to study NCAM-initiated FGFR-mediated signalling, and as a pharmacological compound mimicking *in vivo* effects of NCAM-induced FGFR activation. Indeed, a number of studies report stimulatory effects of FGL on synaptogenesis and memory consolidation [17], survival of hippocampal neurons after ischemic insult [153], and early postnatal sensorimotor development and social memory retention [144]. Recently, FGL also was shown to strongly reduce signs of β-amyloid-induced neuropathology and cognitive impairment in rats [77].

Interestingly, when assessed using surface plasmon resonance analysis, only a double NCAM F3,1-2 module bound to FGFR, but not to the F3,2 module alone [72]. This might mean that the F3,1 module of NCAM also possessed the FGFR-binding site and thus contributed to the NCAM-FGFR interaction. This hypothesis has been tested by Anderson *et al.* [3], who have identified a peptide in the F3,1 module of NCAM (FRM) simulating FGFR activation, as well as FGFR-dependent neurite outgrowth and cell survival. The authors suggested that FRM might represent part of an FGFR activation site of NCAM and may have useful therapeutic applications.

## CONCLUSIONS AND PROSPECTS

Recent advances in characterizing human and other species genomes lead to cloning of a great number of new cell adhesion molecules (see e.g. [178]). It is now widely recognized that these molecules play major roles in almost every physiological and pathological process in multicellular organisms. It is therefore of immense importance to develop reliable pharmacological tools to study specific aspects of CAM functions. However, several intrinsic properties of CAMs make this task particularly challenging. Extracellularly, CAMs are in most cases involved in multiple and relatively low affinity interactions with themselves and with other CAMs and/or cell surface receptors, and the majority of CAMs does not possess any intrinsic enzymatic activity. The first pharmacological tools developed were antibodies targeting specific CAM epitopes. The second generation tools were recombinantly produced protein fragments (modules). Both approaches have a major drawback, the antibodies and the recombinant fragments are relatively large, unstable proteins, which makes it particularly difficult to use them for *in vivo* studies. The third, peptide-based, generation of pharmacological tools came along with the progress in our understanding of the structural basis of CAM-mediated cell adhesion allowing structure-based design of CAM mimetic peptides. The peptide ligands of CAMs can target particular regions of the complex CAM interaction sites and induce specific signalling pathways, thereby allowing one to mimic specific aspects of CAM functions. In addition, these peptide mimetics are potential therapeutic agents. The therapeutic use of peptides is believed to be limited by their generally poor penetration through the blood-brain barrier (BBB). However, a recent study [144] indicates that a peptide from the NCAM binding site for FGFR, FGL, rapidly penetrates into the blood and cerebrospinal fluid after both intranasal and subcutaneous administration and remains detectable in the fluids for up to 5 hours. In addition, modifications of the peptides can be made to facilitate crossing the BBB. Such modifications may include, for example, conjugation of the two peptides, where the first represents an 'active' sequence, and the second is known to cross the BBB by binding to appropriate 'carrier' receptors, such as megalin or LRP. Since peptides derived from low-affinity binding sites are by default the low-affinity ligands, the next step in developing CAM-targeted pharmacological tools will be the generation of low molecular weight non-peptide molecules with high-affinity binding properties. The structural basis of cell adhesion is a rapidly developing field, and the latter probably may be achieved through homology modelling and molecular docking.

## Figures and Tables

**Fig. (1):**
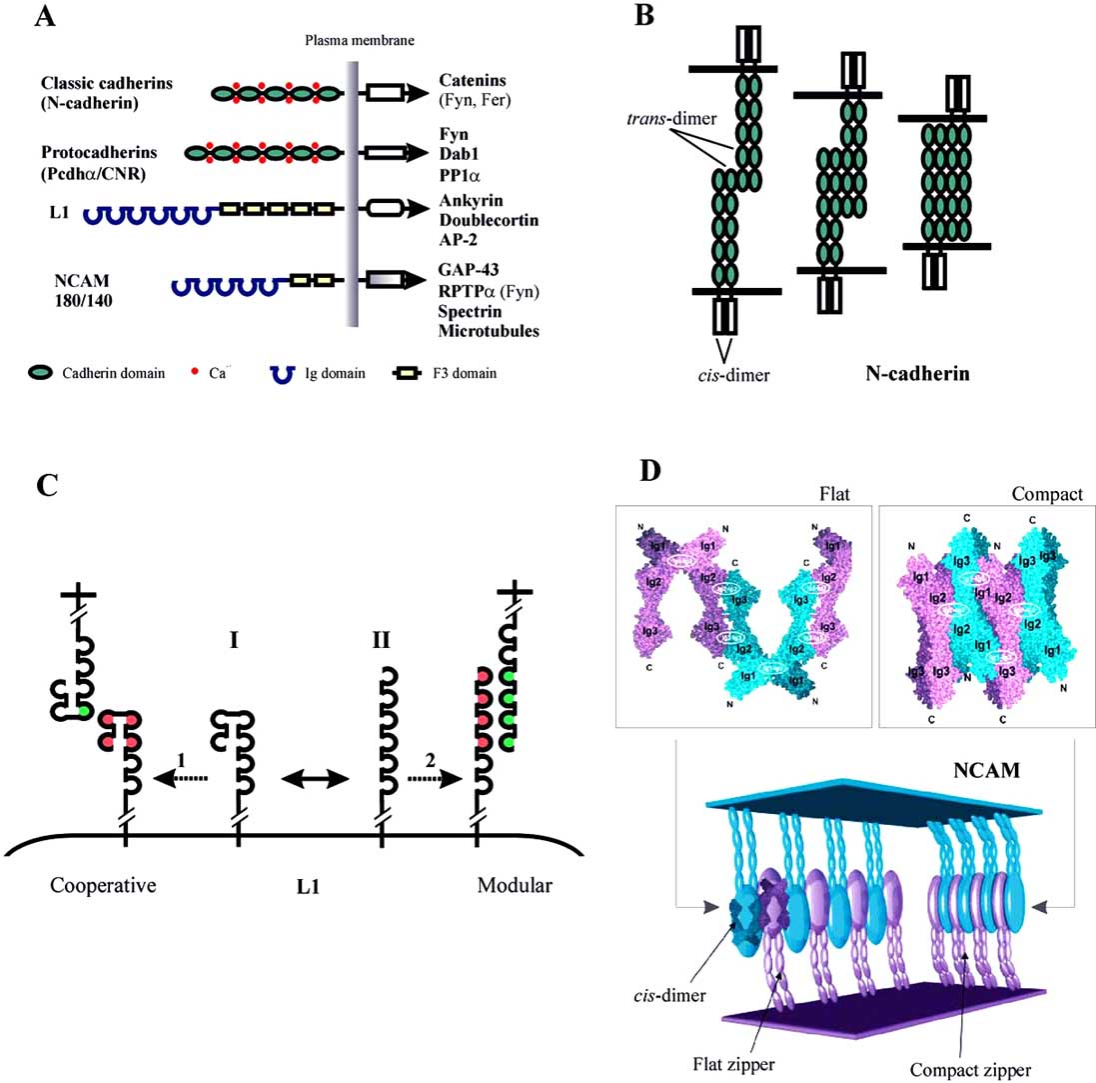
Structural basis of CAM-mediated cell adhesion. (A)Domain structure of classic and proto- cadherins, L1, and NCAM 180/140. Binding partners for cytoplasmic tails of CAMs are shown. (B) A model of cadherin-mediated cell adhesion (shown for N-cadherin). (C) Two proposed mechanisms of homophilic L1 binding. Note that the cartoon of L1 is shortened to highlight Ig1-Ig6. The horseshoe (I) and the extended (II) conformations of L1 exist in dynamic equilibrium and may underlie two different mechanisms of homophilic L1 binding, occurring in the cooperative (1) or modular (2) mode. See text for details. (D)A model of homophilic NCAM binding. Top: crystal structure of NCAM Ig1-Ig2-Ig3 showing interaction between the three Ig-modules. Bottom: organization of the extracellular part of NCAM molecules engaged in both a flat and a compact zipper. The large ellipsoids correspond to the two interacting Ig1-Ig2-Ig3 constructs as shown in the leftmost part (modified from [73]).

**Fig. (2):**
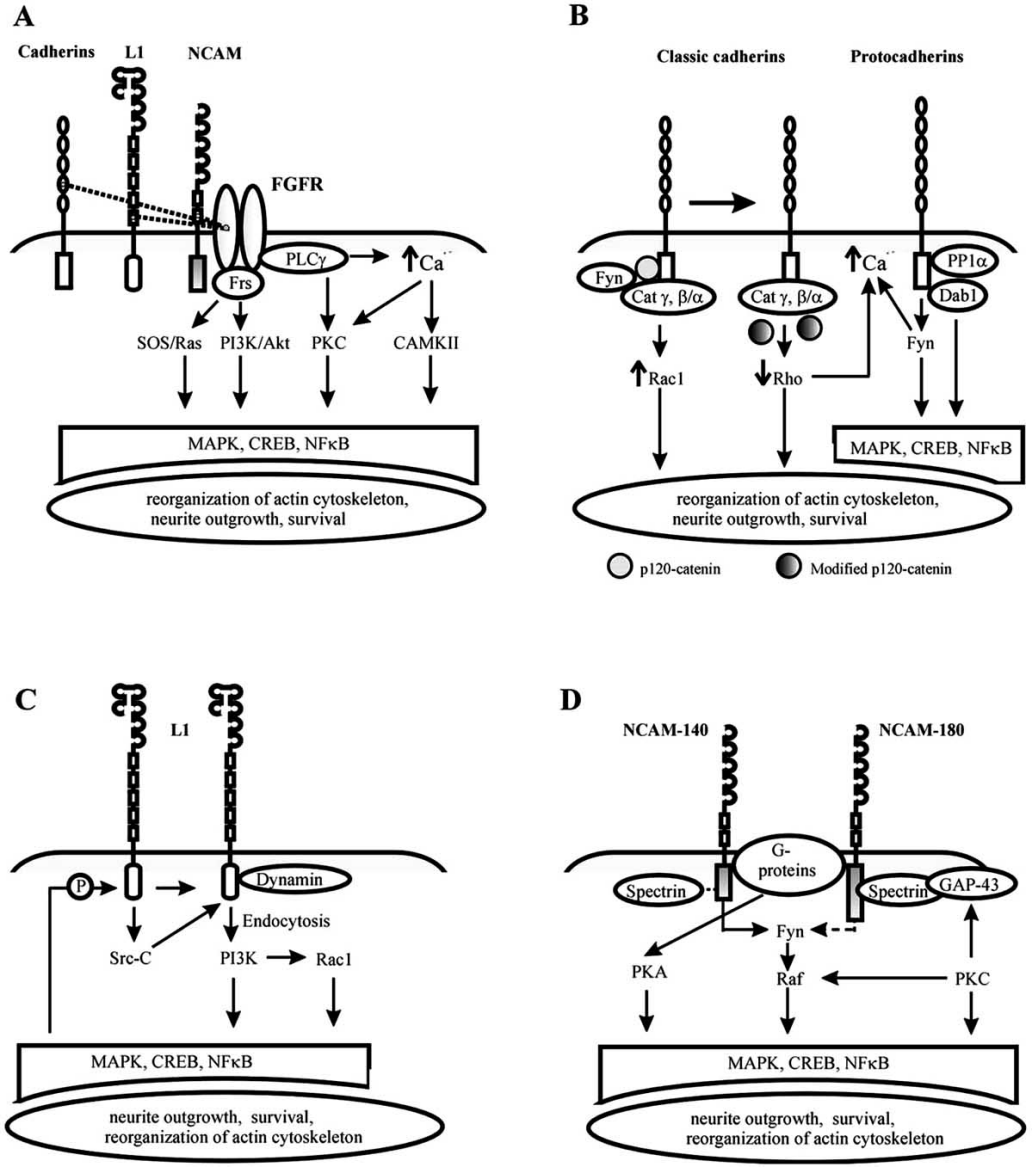
Intracellular signalling by CAMs. Signal transduction pathways associated with FGFR (A) and cytoplasmic parts of cadherins (B), L1-CAM (C), and NCAM (D). Stippled arrows indicate weak interactions and/or putative signalling links. See text for details.

**Table 1:** Pharmacological Tools to Study CAM Function

Pharmacological Approach	Selected Studies
**Cadherins:** 'Ca^2+^-switch' method [112,21] Recombinant cadherin fragments [13,91,85] Monitoring the 2D movement of cadherin-coated beads [90,157] Synthetic peptides	Role of cadherins in axonal growth and targeting [121,48] Role of cadherins in formation of cell junctions and postsynaptic density [181,56] Cadherin-induced modulation of LTP [191] Identification of an N-cadherin motif interacting with FGFR [185,188] Cadherin-mediated adhesion [13] and lateral clustering [193] Cadherin-induced neurite outgrowth [173] Recruitment of catenins and regulation of cytoskeleton by cadherins [85,91] Time-course of cadherin-induced modulation of Rac and Rho [112,21] Functional link between the cytoplasmic domain of N-cadherin and voltage-gated Ca^2+^ channels [118] Anchoring of cadherins to the cytoskeleton [137,91,170] Identification of peptide inhibitors [186,187] and activators [189,151] of cadherin function
**L1-CAM:** Inhibitory antibodies Activating antibodies Recombinant L1 proteins Chromophore-assisted laser inactivation of L1 [180] Synthetic peptides	Role of L1 in cell migration [97] and aggregation [125,68] Interactions L1:axonin-1 [87] and L1:NCAM [57] Role of L1 in neurite outgrowth/fasciculation [42,43] and Schwann cell differentiation [145] Triggering of intracellular signalling by L1 [78,141,39] Involvement of phosphatases and MAP kinases in L1-induced signalling [78,141,166] Mimicking of neurite- and survival-promoting effects of L1 *in vitro* [399] Role of FGFR in the L1-induced neurite outgrowth [37] Signalling pathways involved in the L1-induced neuroprotection [99] Role of individual modules of L1 in the L1:integrin binding [113,148] and in the L1:neuropilin-1 binding [19] Mapping the homophilic binding domain in L1 [197,52] L1-induced acceleration of nerve regeneration *in vivo* [133,190] The role of L1 in growth cone protrusion and neurite extension [161] Identification of peptide inhibitors [57,198] of L1 function
**NCAM:** NCAM-specific antibodies Recombinant NCAM proteins Chromophore-assisted laser inactivation of NCAM [180] Enzymatic removal of NCAM-associated PSA Synthetic peptides	NCAM-mediated cell adhesion [12,135] Role of NCAM in neurite growth, guidance and fasciculation [136,93] Isolation of PSA-binding regions of NCAM [24] Mapping of functionally important sites of NCAM [47] Role of NCAM in neuronal migration [143] and long-term potentiation [100,128] Role of GAP-43 in the NCAM-induced neurite outgrowth [104] Elucidation of the mechanism of homophilic NCAM binding [25,155] Role of individual modules of NCAM in different cell functions [46,123] The role of NCAM in growth cone protrusion and neurite extension [161] Role of PSA-NCAM in axonal growth and branching [34,196], cell migration [74,6], and synaptic plasticity [107,33] Identification of PSA-derived peptides promoting neuronal differentiation and migration [171], and improving long-term memory [44] Identification of peptides mimicking NCAM function [129,76,154,116,132,72,3,8] and possessing neuroprotective and memory consolidating effects *in vivo* [17,153,77]

## ABBREVIATIONS

2-AG: = 2-arachidonoyl glycerol
AA: = Arachidonic acid
(N)CAM: = (Neural) cell adhesion molecule
CaMK: = Calcium-calmodulin kinase
CREB: = Cyclic AMP response element binding protein
Dab1: = Disabled 1
DAG: = Diacylglycerol
ECM: = Extracellular matrix
ERK: = Extracellular signal-regulated kinase
F3: = Fibronectin type III
FGFR: = Fibroblast growth factor receptor
GAP-43: = Growth-associated protein-43
GPI: = Glycosylphosphatidyl inositol
Ig: = Immunoglobulin (like)
IP3: = Inositoltrisphosphate
LTP: = Long-term potentiation
MAPK: = Mitogen activated protein kinase
MASA: = Mental retardation, aphasia, shuffling gait (syndrome)
MEK: = MAPK/ERK kinase
NP-1: = Neuropilin-1
NSCC: = Non-selective cation channel
PAK: = p21-activated kinase
PDK: = Phosphoinositide-dependent protein kinase
PI3K: = Phosphatidylinositol 3-kinase
PKC: = Protein kinase C
PLC: = Phospholipase C
PP1α: = Serine/threonine protein phosphatase 1α
PSA: = Polysialic acid
RTK: = Receptor tyrosine kinase
SP1: = Spastic paraplegia type 1
TRP: = Transient receptor potential (channel)(channel)
VDCC: = Voltage-dependent Ca^2+^channel
